# Supplementary material for: The protein translation machinery is expressed for maximal efficiency in Escherichia coli
Source: Nat Commun. 2020 Oct 16;11:5260. doi: 10.1038/s41467-020-18948-x (PMC7568582; doi:10.1038/s41467-020-18948-x)
Supplement: Supplementary file 1 — Supplementary Information [file 41467_2020_18948_MOESM1_ESM.pdf]

The protein translation machinery is expressed for maximal efficiency in *Escherichia coli*

Hu *et al.*

This PDF includes:

Supplementary Notes 1 - 3

Supplementary Tables 1 - 5

Supplementary Figures 1 - 8

Supplementary References

Supplementary Notes

1. The coarse-grained optimization models by Scott *et al.* and Klumpp *et al.*

In Ref. [1], Scott *et al.* use a phenomenological model based on the „bacterial growth laws" most prominently described in Ref. [2]. This model considers two proteome sectors, a ribosomal sector with proteome fraction Φ_R and a metabolic sector with proteome fraction Φ_P, responsible for the production of the amino acids consumed by the ribosomal sector. A constraint relates these two proteome sectors to the maximally available proteome fraction for protein synthesis, Φ_R^{max}, which is assumed to be constant: $\Phi_R + \Phi_P = \Phi_R^{max}$. Thus, the model of Scott *et al.* has only one free parameter, the proteome fraction allocated to the ribosomal sector, Φ_R. For a given Φ_R , the growth rate (which is defined by the rate of protein production) is set by three phenomenological parameters: the "translational efficiency" γ (translation rate per ribosomal proteome fraction), assumed to be constant; the "nutritional efficiency" ν (amino acid production rate per metabolic proteome fraction), assumed to be condition-dependent; and a constant proteome fraction of inactive ribosomes, Φ_R^{min} . Maximizing the growth rate under these constraints results in an optimal ribosomal proteome fraction.

The approach by Scott *et al.* aims to answer broadly the same question as explored in the present work: given that cellular resources are limited, what is the optimal way to allocate them in order to allow fast growth? Scott *et al.* approach this question by maximizing the growth rate while assuming constant translational efficiency γ and fraction of inactive ribosomes Φ_R^{min}. The parameters are derived from fits to coarse-grained experimental data. With this approach, Scott *et al.* show that under relatively simple assumptions, an optimal allocation of proteome mass to translation and metabolism exists, and the relationship between the ribosomal proteome fraction and the growth rate is qualitatively similar to that observed experimentally, i.e., is linear. While in subsequent publications of the same group, the model of Scott *et al.* has been shown to be very powerful at explaining growth-related phenomena, it requires parameters fitted to experimental data, and the mechanistic basis of its components are unclear. In particular, there is no clear explanation for the existence and size of the "offset" of the ribosomal proteome fraction at zero growth rate in this model, Φ_R^{min}.

While our model minimizes the cost of translation rather than maximizing growth rate, our approach is mathematically equivalent to a maximization of growth rate under a constraint on the total cost and under certain additional assumptions (such as a constant amino acid composition of the proteome across growth rates). Both our approach and that of Scott *et al.* vary some condition-dependent parameters (the nutritional efficiency for Scott *et al.*, the proteome mass and composition in our manuscript) and then optimize an aspect of cellular resource allocation. However, in contrast to Scott *et al.*, we are not interested in the relative global resource allocation between translation and biosynthesis based on a schematic, coarse-grained model, but in a mechanistic explanation of the quantitative pattern of resource allocation across different components of the translation machinery.

Building on the same phenomenological bacterial growth laws [2] as Scott *et al.* [1], Klumpp *et al.* [3] also analysed the composition of the translation machinery. Noting that this machinery includes not only ribosomes, but also other highly expressed proteins – most notably elongation factors [4] and tRNA synthetases – Klumpp *et al.* argued that a full appreciation of the efficiency of protein synthesis requires the inclusion of the cost of these translation components. They extended the phenomenological, coarse-grained model of Ref. [2] into four proteome sectors, including a ribosomal (Rb) and a translation-associated (T) sector. Assuming co-regulation of the Rb and T sectors and fitting three phenomenological constants to the data, they were able to approximate the growth-rate dependence of ribosome concentration and elongation speed in *E. coli* [3]. However, the experimentally

observed ratio between the protein concentrations in the T- and Rb-sectors, Φ_T/Φ_{Rb} , deviates from the postulated constant ratio (see Fig. 3D in Ref. [3]), indicating shortcomings of this phenomenological theory.

Klumpp *et al.* also attempted to determine an optimal growth-rate dependence of the ratio between T- and Rb-sectors, by treating both proteome fractions as independent parameters when numerically optimizing the growth rate of their coarse-grained model cell. However, the results predicted a ratio Φ_T/Φ_{Rb} that was substantially smaller than that observed (see Fig. 4C in Ref. [3]), indicating that translation in *E. coli* is either not organized optimally, or that the objective optimized by natural selection differs from the proteome allocation examined by Klumpp *et al.*. Comparing the objective functions used by Klumpp *et al.* and in the present work, we note that proteins make up 1/3 of the ribosome, but 2/3 of the ternary complex (by mass). Thus, the ternary complex appears much more expensive to the cell when considering protein mass than when considering total mass, explaining why optimization of protein allocation results in smaller predictions of the Φ_T/Φ_{Rb} ratio [3,5].

2. Ribosome states

The ribosome is the most central component of translation, and the ribosome states in our model are slightly different from those used in proteome partitioning models [2,3,6]. In this section, we will discuss the difference and the rationality of ribosome states in our model.

Briefly, our model contains active ribosomes and free ribosomes. **Active ribosomes** are bound to mRNA and actively involved in elongating peptide chains. **Free ribosomes** are responsible for translation initiation; they are available for binding to mRNA and comprise a subset of the inactive ribosomes in proteome partitioning models.

Proteome partitioning models distinguish between active ribosomes and inactive ribosomes. **Active ribosomes** have exactly the same meaning as in our model: they are engaged in elongation. At steady-state growth, the protein synthesis rate can be written as: $v_{protein\_syn} = \mu P = f_{active} \cdot k_{eff} \cdot R$, where μ is the growth rate, P is the total protein concentration (measured in amino acids per volume), f_{active} is the fraction of active ribosomes among total ribosomes, k_{eff} is the turnover number of ribosomes during elongation, and R is the concentration of ribosomes. By measuring μ, k_{eff}, and the ratio between R and P (estimated through the RNA/Protein ratio and the fraction of rRNA in total RNA), Dai *et al.* estimated the fraction of active ribosomes as a function of growth rate [6]. In the view of protein partitioning models, the **inactive ribosomes** comprise all ribosomes not actively engaged in elongation. Inactive ribosomes include not only ribosomes available for initiation (**free ribosomes**), but also ribosomes that are unavailable for initiation (**unused, or deactivated, ribosomes**) [2,3,6]. In this work, we modeled both initiation and elongation, and thus both free and active ribosomes (but not deactivated ribosomes) are included.

Our model is carefully built on first principles. All reactions are explicitly and exclusively constrained by reaction parameters and steady state growth; we avoid any empirical growth rate-dependent parameters, such as a growth rate-dependent fraction of active ribosomes or effective ribosome activity. In other words, our model contains only reactions for which we know why and how they occur. The mechanism leading to a fraction of deactivated ribosomes is not clear. Deactivated ribosomes facilitate faster transitions between growth environments that support different growth rates [7], a phenomenon that cannot be predicted with steady-state models such as ours. Moreover, the true fraction of deactivated ribosomes has not been measured experimentally. Thus, we did not attempt to predict the total concentration of ribosomes (including deactivated ribosomes), and only compared our predictions for active ribosome concentrations to experimental estimates.

3. Impact of GTP and GDP concentrations on the predictions

GTP and GDP are involved in many intracellular reactions [8], and we thus do not expect to predict their concentrations in this translation model. In our model, GTP and GDP are involved in nucleotide exchange by elongation factor Tu [9] (see Methods). The concentrations of GTP and GDP may influence the rates of some reactions directly. In this section, we assess the impact of the assumed GTP and GDP concentrations on the predictions, examining three pairs of concentrations [10] resulting from growth of *E. coli* K-12 on different media. Note that GTP and GDP concentrations [10] and proteome data were collected for different strains of *E. coli* K-12 (NCM3722 and BW25113, respectively).

The GTP/GDP measurements were done for growth on acetate (c_{GTP} = 1250 µM; c_{GDP} = 18 µM), glycerol (c_{GTP} = 2690 µM; c_{GDP} = 23 µM), and glucose (c_{GTP} = 4900 µM; c_{GDP} = 680 µM); all three conditions also appear in our simulations. We first simulated growth on acetate and on glycerol with GTP and GDP concentrations measured for *E. coli* cells growing on the same media. Next, we replaced the GTP and GDP concentrations with the data for glucose and repeated the simulations (Supplementary Fig. 8). Despite the large differences in GTP and GDP concentrations, the results obtained are very similar. For both acetate and glycerol growth, geometric mean fold-errors (GMFE) are below 1.03 (Supplementary Fig. 8), *i.e.*, the predicted concentrations of the individual components of the translation machinery are on average less than 3% higher or lower in the two sets of predictions. Thus, GTP and GDP concentrations appear to have only a minor influence on the predictions. Because glucose is the reference condition for the protein expression data [4], we used the concentration of GTP and GDP for growth on glucose for all predictions in this study.

Supplementary Figures

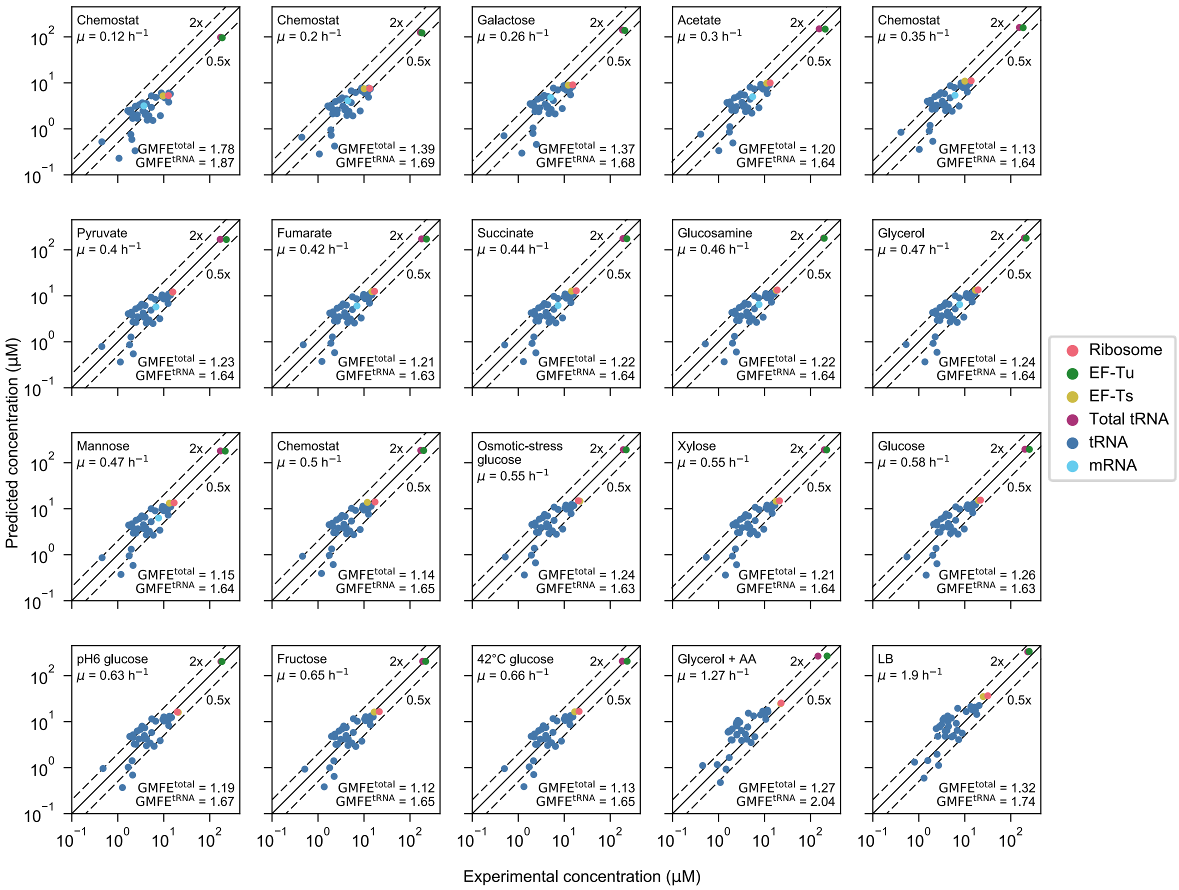

Supplementary Figure 1. The predicted optimal concentrations of the components of the translation machinery agree with experimental estimates across 20 growth conditions on different media and in chemostats with a minimal glucose medium (sorted by ascending growth rate). The conditions are those under which protein concentrations were measured in Ref.[4]. mRNA[11] and tRNA[12] were assayed in conditions with growth rates that differ from those of the proteomics experiment. To plot mRNA and tRNA data in the same panels, we fitted second order polynomial regression models to the available data for mRNA and tRNA concentrations, respectively, and then used the regressions to estimate the concentrations at the growth rates shown in the panels. Absolute mRNA concentration[11] was only assayed for growth rates between 0.11 h^{-1} and 0.49 h^{-1}, and we did not attempt to extrapolate mRNA concentrations beyond this range.

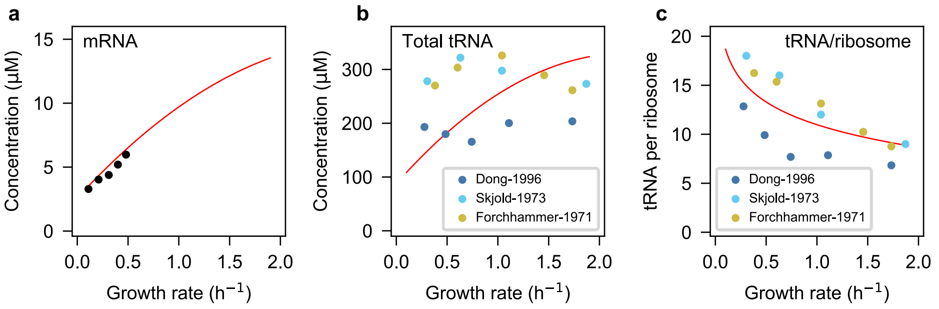

Supplementary Figure 2. The concentrations of the major non-ribosomal RNA pools predicted from cost minimization are consistent with experimental observations. (a) mRNA [11], R^2 = 0.97, GMFE = 1.06. **(b)** Total tRNA data from Dong *et al.* [12] (summed over individual tRNAs), Forchhammer *et al.* [13], and Skjold *et al.* [14]; combined R^2 = 0.27, GMFE = 1.30. (**c**) number of tRNAs per ribosome from the same datasets as in (b).

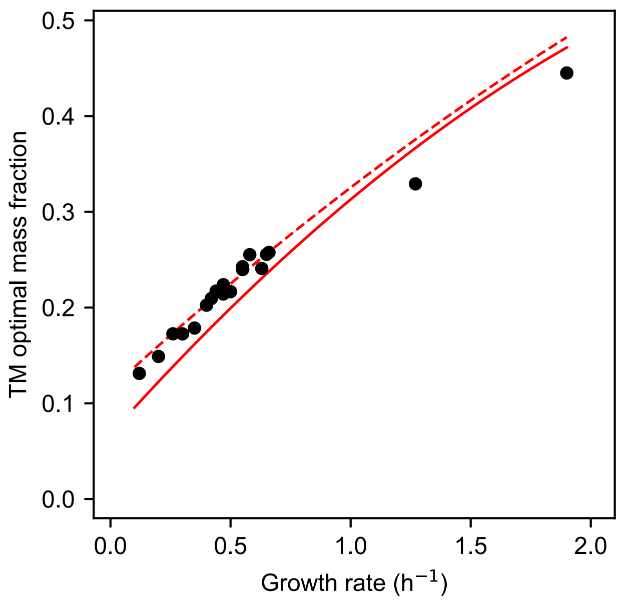

Supplementary Figure 3. Theoretically optimal resource allocation to the translation machinery as a fraction of total dry mass increases almost linearly with growth rate. The solid red line indicates the model predictions, without accounting for deactivated ribosomes. The dashed line indicates the predicted optimal mass fraction when we additionally include the fraction of deactivated ribosomes, which cannot be predicted by a steady-state model but which we estimated from experimental observations (Fig. 4 of the main text; see Methods for details). Experimental data (points) is the sum over the observed concentrations of translation associated proteins [4] (ribosomal proteins, EF-Tu, EF-Ts) and RNA [2,6] (ribosomal RNA, tRNA, mRNA; interpolated to the same growth rates as in the protein measurements, see Methods). Note that the mass fraction of the translation machinery does not include GDP, GTP, free tRNA, tRNA-synthetases, and elongation factor G (FusA).

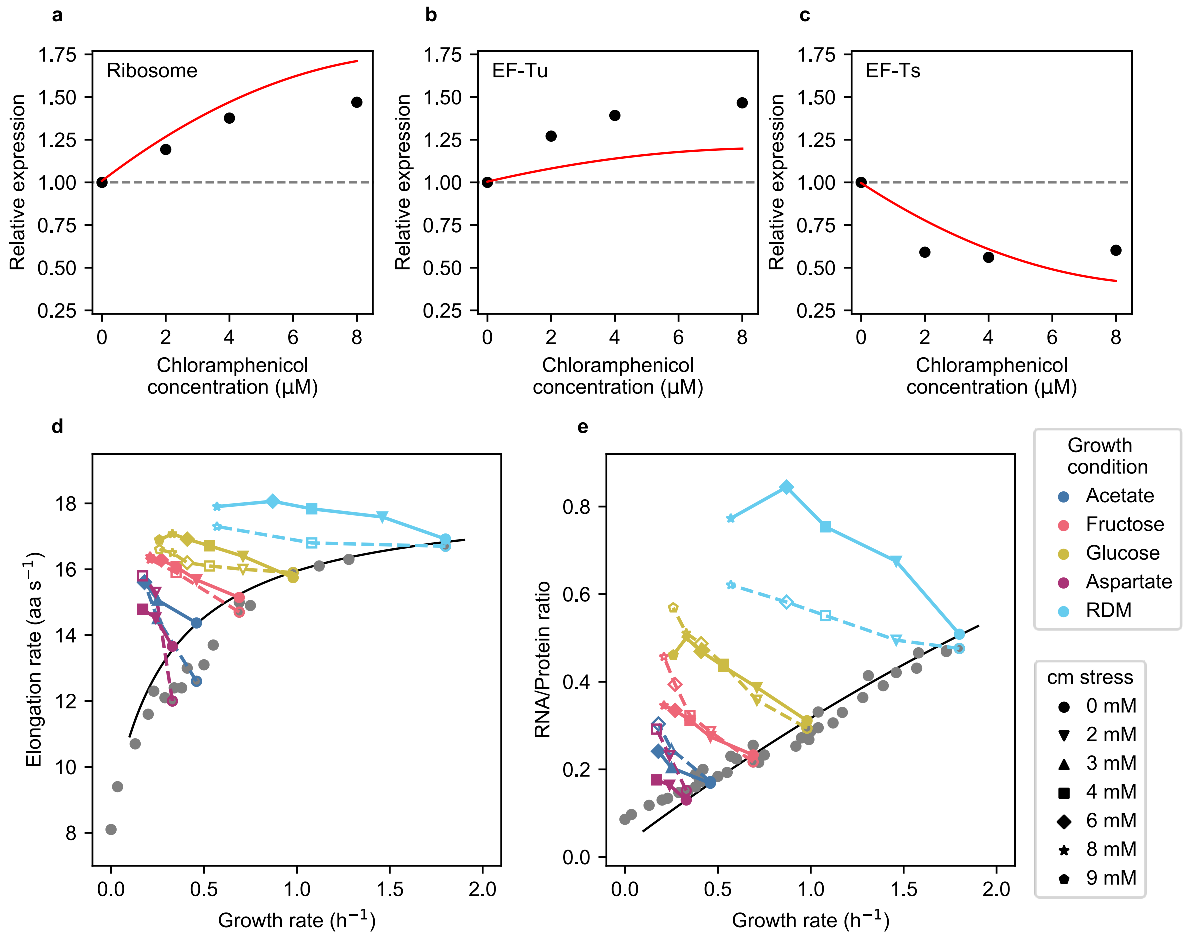

Supplementary Figure 4. Optimality of the translation machinery under chloramphenicol stress. Model predictions (red lines) of relative changes in the concentrations of **(a)** ribosome, **(b)** EF-Tu, and **(c)** EF-Ts under increasing chloramphenicol stress are qualitatively consistent with experimental data [15] [**a**, **b**, **c** show the results for growth on glucose]. Predicted **(d)** elongation rates and **(e)** RNA/protein ratios under chloramphenicol stress are also qualitatively consistent with experimental data [6]. Grey dots indicate experimental elongation rates without chloramphenicol stress; the black line marks the corresponding (non-stressed) predictions. Different symbols indicate varying chloramphenicol concentrations, while colours indicate growth conditions (different nutrients). Dashed lines connect experimental elongation rates (open symbols) under chloramphenicol stress on the same nutrient; solid lines connect the corresponding elongation rate predictions (filled symbols). Chloramphenicol concentrations were varied from 0 mM to 9 mM. In both predictions and experiment, elongation rates increase with growing chloramphenicol stress, with faster increases under progressively poorer nutrient conditions. The overestimated RNA/protein ratio on rich defined medium (RDM) likely reflects the fact that ribosome is inhibited less by chloramphenicol *in vivo* than theoretical calculations predict (see Fig. N1 in Ref. [6]). The predictions are functions of the growth rate and of chloramphenicol concentration; the non-smoothness of the prediction lines likely arise from experimental uncertainties in the corresponding values.

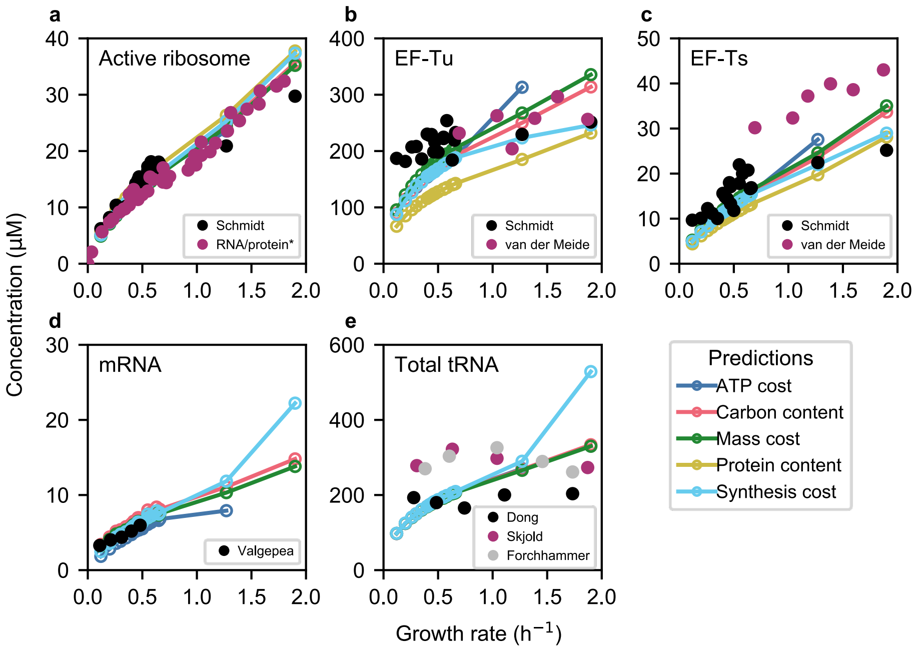

Supplementary Figure 5. Different cost definition lead to broadly similar growth rate dependencies. The coloured lines show predictions based on minimizing the total mass density (as in the main text), carbon content, protein mass, and synthesis cost of the components of the translation machinery, respectively. The panels compare the predictions to experimental estimates for (**a**) active ribosomes (based on proteomics [4], black dots, and RNA/protein ratios, red dots); (**b**) EF-Tu; (**c**) EF-Ts; (**d**) mRNA; and (**e**) total tRNA. As it is unclear how to calculate ATP costs in the LB medium (μ = 1.9 h^{-1}), no results for ATP costs are shown for this condition.

To derive the molar concentration of active ribosomes from reported RNA/protein mass ratios for panel (a), we used the tRNA/ribosome ratios reported in the experimental papers on tRNA concentrations [12–14] to calculate the rRNA in total RNA (f_{rRNA}) (as mRNA is a very small fraction of total RNA by mass (~ 2-5%), we ignored its contribution in this calculation). With f_{rRNA}, the RNA/Protein mass ratio [2,6], and the mass fraction of protein in the ribosome, we calculated the fraction of ribosomal protein in total protein. Then, with equation (1) and the equation for the active ribosome fraction ($f_{active} = \mu / (0.124 + \mu)$) we calculated the active ribosome concentration.

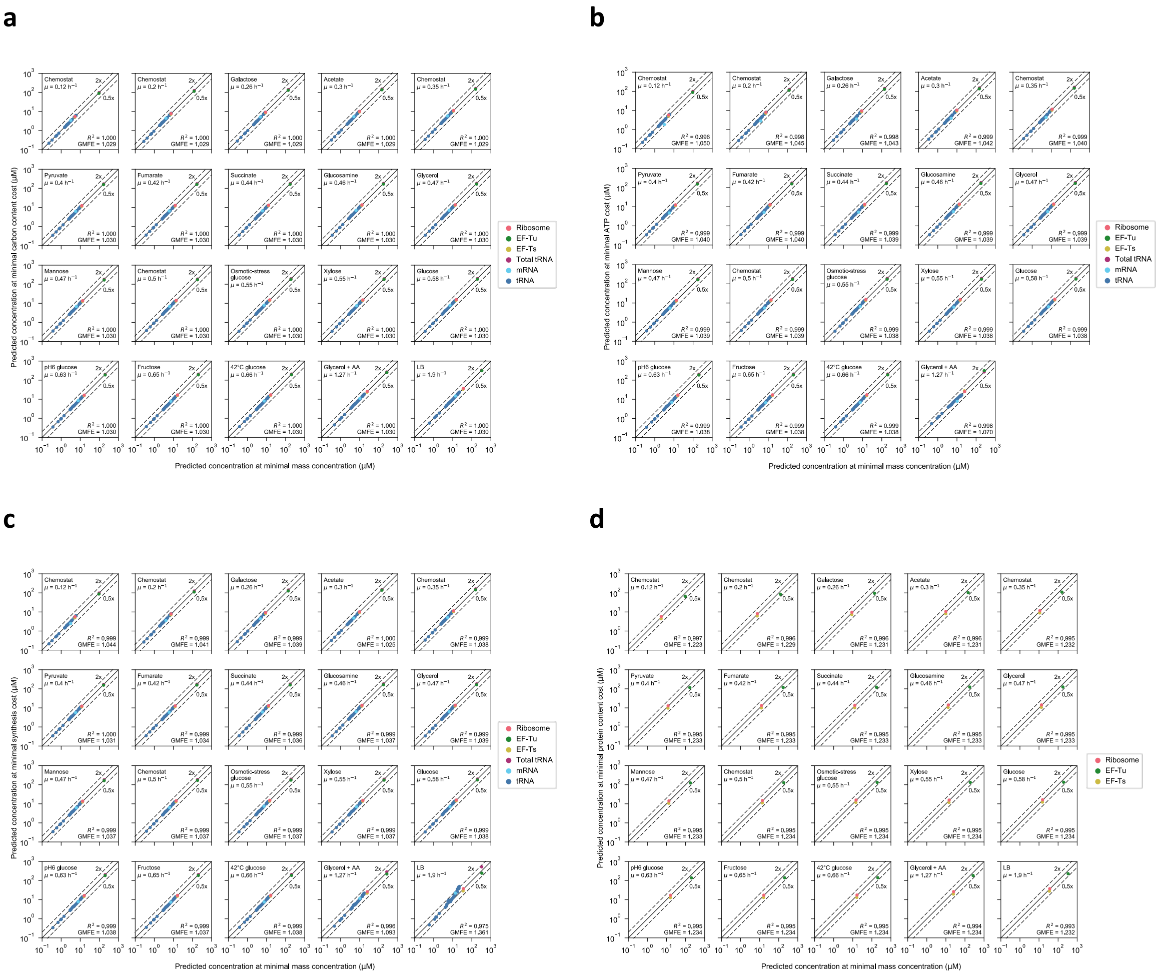

Supplementary Figure 6. Comparison of predicted concentrations at minimal mass concentration with alternative cost measures. Alternative cost measures based on **(a)** carbon content, **(b)** ATP cost of synthesis, and **(c)** the macromolecular investment into the synthesis lead to very similar predictions of the concentrations of the translation machinery components as mass concentration costs. A cost measure based on the protein content **(d)** does not assign costs to mRNA and tRNA and can hence not predict their concentrations. Each sub-panel corresponds to one growth condition assayed in Ref. [4]. A data point shows the predicted concentration for one component based on an alternative cost measure vs. the predicted concentration based on the mass concentration cost employed for Figures 2-5 in the main text. In the bottom right corner of each sub-panel, we provide the square of Pearson's correlation coefficient on log-scale, R^2, and the geometric mean fold error, GMFE. As it is not clear how to estimate ATP costs in the rich medium (LB), we made no predictions for this condition in (b).

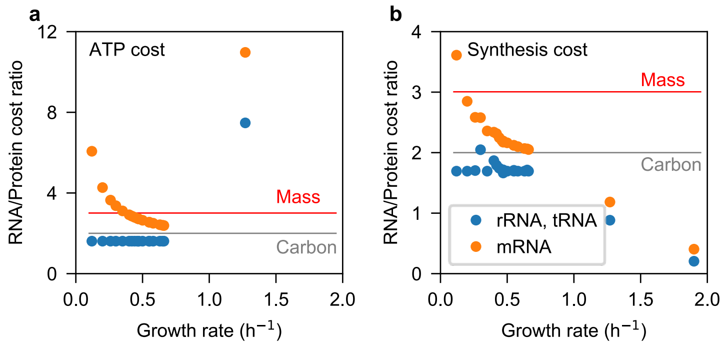

Supplementary Figure 7. The cost of RNA per nucleotide, divided by the cost for the synthesis of protein per amino acid, plotted against the growth rate μ. (a) ATP cost of synthesis; **(b)** total required catalyst mass of synthesis (synthesis cost). The horizontal red line shows the RNA/protein cost ratio for the mass concentration cost, the horizontal grey line the cost ratio based on the carbon content. For ATP and synthesis costs, the RNA/protein cost ratios are different between stable RNA (tRNA, rRNA) and mRNA, as for mRNA we additionally consider degradation. The row of identical rRNA, tRNA cost ratios at low growth rates in (b) is for chemostat conditions with a minimal glucose medium.

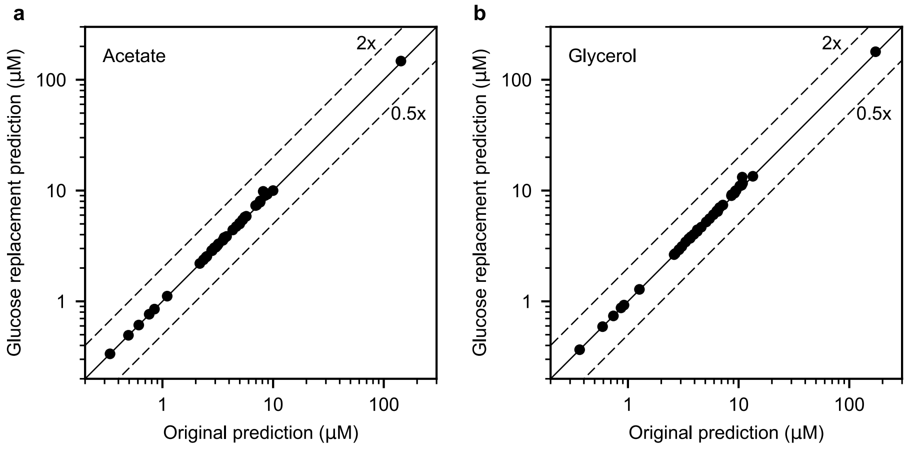

Supplementary Figure 8. Impact of GTP and GDP concentrations on model predictions. (a) Growth on acetate (geometric mean fold-error GMFE = 1.028). **(b)** Growth on glycerol (*GMFE* = 1.030). Each datapoint represents the concentration of one model component (ribosome, EF-Tu, EF-TS, aa-tRNA). x-axes show predictions using the GTP and GDP concentrations measured for the corresponding medium; y-axes show predictions when instead assuming the GTP and GDP concentrations measured for growth on glucose. GMFE measures the mean deviation from the identity line on the log-log plot; GMFE = 1 indicates perfect identity. The very low GMFE values indicate that *in vivo* GTP and GDP concentration has a very small effect on our model.

Supplementary Tables

Supplementary Table 1. tRNAs - anticodon, cognate codons, common name(s), and corresponding genes.

tRNA in the model	Anticodon	codon(s)	tRNA(s) in *Dong et al* [12]	Corresponding genes
Ala1B	UGC	GCU, GCA, GCG	Ala1B	alaV, alaU, alaT
Ala2	GGC	GCC	Ala2	alaX, alaW
Arg2	ACG	CGU, CGC, CGA	Arg2	argQ, argZ, argY, argV
Arg3	CCG	CGG	Arg3	argX
Arg4	UCU	AGA	Arg4	argU
Arg5	CCU	AGG	Arg5	argW
Asn	GUU	AAC, AAU	Asn	asnT, asnW, asnU, asnV
Asp1	GUC	GAC, GAU	Asp1	aspU, aspV, aspT
Cys	GCA	UGC, UGU	Cys	cysT
Gln1	UUG	CAA	Gln1	glnW, glnU
Gln2	CUG	CAG	Gln2	glnX, glnV
Glu2	UUC	GAA, GAG	Glu2	gltW, gltU, gltT, gltV
Gly1	CCC	GGG	Gly1	glyU
Gly2	UCC	GGA, GGG	Gly2	glyT
Gly3	GCC	GGC, GGU	Gly3	glyW, glyV, glyX, glyY
His	GUG	CAC, CAU	His	hisR
Ile1	GAU	AUC, AUU	Ile1	ileV, ileY, ileU, ileT
Ile2	CAU	AUA	Ile2	ileX
Leu1	CAG	CUG	Leu1	leuT, leuV, leuP, leuQ
Leu2	GAG	CUC, CUU	Leu2	leuZ
Leu3	UAG	CUA, CUG	Leu3	leuU
Leu4	CAA	UUG	Leu4	leuW
Leu5	UAA	UUA, UUG	Leu5	leuX
Lys	UUU	AAA, AAG	Lys	lysT, lysW, lysY, lysZ, lysQ, lysV
Met	CAU	AUG	Met m	metU, metT
Phe	GAA	UUC, UUU	Phe	pheV, pheU
Pro1	CGG	CCG	Pro1	proK
Pro2	GGG	CCC, CCU	Pro2	proL
Pro3	UGG	CCA, CCU, CCG	Pro3	proM
Ser1	UGA	UCA, UCU, UCG	Ser1	serT
Ser2	CGA	UCG	Ser2	serU
Ser3	GCU	AGC, AGU	Ser3	serV
Ser5	GGA	UCC, UCU	Ser5	serW, serX
Thr13	GGU	ACC, ACU	Thr1, Thr3	thrV, thrT
Thr2	CGU	ACG	Thr2	thrW
Thr4	UGU	ACA, ACU, ACG	Thr4	thrU
Trp	CCA	UGG	Trp	trpT
Tyr12	GUA	UAC, UAU	Tyr1, Tyr2	tyrV, tyrT, tyrU
Val1	UAC	GUA, GUG, GUU	Val1	valT, valZ, valU, valX, valY
Val2	GAC	GUC, GUU	Val2A, Val2B	valV, valW

Supplementary Table 2. tRNAs.

Codon	tRNA[1]
GGG	Gly1, Gly2
GGA	Gly2
CUG	Leu1, Leu3
CUA	Leu3
UUG	Leu4, Leu5
UUA	Leu5
CCG	Pro1, Pro3
CCC	Pro2
CCU	Pro2, Pro3
CCA	Pro3
UCA	Ser1
UCG	Ser1, Ser2
UCU	Ser1, Ser5
UCC	Ser5
ACC	Thr13
ACU	Thr13, Thr4
ACG	Thr2, Thr4
ACA	Thr4
GUG	Val1
GUA	Val1
GUU	Val1, Val2
GUC	Val2

[1] tRNAs predicted to be non-expressed (concentration 0 µM) are shown in red.

Supplementary Table 3. RNA/protein cost ratios.

Condition	Growth rate (h^{-1})	$\text{RNA/protein cost ratio} = \frac{\text{RNA cost per NTP}}{\text{protein cost per amino acid}}$					
		Mass	Carbon content	ATP cost[1] (sRNA/AA)	ATP cost (mRNA/AA)	Synthesis cost[1] (sRNA/AA)	Synthesis cost (mRNA/AA)
42°C glucose	0.66	3.01	2.00	1.61	2.39	1.69	2.05
Acetate	0.30	3.01	2.00	1.61	3.37	2.05	2.58
Chemostat μ = 0.12	0.12	3.01	2.00	1.61	6.07	1.69	3.61
Chemostat μ = 0.2	0.20	3.01	2.00	1.61	4.27	1.69	2.85
Chemostat μ = 0.35	0.35	3.01	2.00	1.61	3.11	1.69	2.36
Chemostat μ = 0.5	0.50	3.01	2.00	1.61	2.65	1.69	2.16
Fructose	0.65	3.01	2.00	1.61	2.40	1.71	2.06
Fumarate	0.42	3.01	2.00	1.61	2.85	1.79	2.31
Galactose	0.26	3.01	2.00	1.61	3.64	1.70	2.58
Glucosamine	0.46	3.01	2.00	1.61	2.74	1.71	2.20
Glucose	0.58	3.01	2.00	1.61	2.50	1.69	2.10
Glycerol	0.47	3.01	2.00	1.61	2.72	1.66	2.17
Glycerol + AA	1.27	3.01	2.00	7.48	10.97	0.88	1.18
LB	1.90	3.01	2.00	NA	NA	0.21	0.40
Mannose	0.47	3.01	2.00	1.61	2.72	1.72	2.19
Osmotic-stress glucose	0.55	3.01	2.00	1.61	2.55	1.69	2.12
pH6 glucose	0.63	3.01	2.00	1.61	2.42	1.69	2.07
Pyruvate	0.40	3.01	2.00	1.61	2.92	1.87	2.34
Succinate	0.44	3.01	2.00	1.61	2.79	1.74	2.25
Xylose	0.55	3.01	2.00	1.61	2.55	1.71	2.12

[1] sRNA = rRNA and tRNA

Supplementary Table 4. The protein, stable RNA (tRNA and rRNA), and mRNA synthesis costs (results from ccFBA).

Condition	Growth rate (h^{-1})	v_{AA} (mmol·gDW^{-1}·h^{-1})	v_{sRNA} (mmol·gDW^{-1}·h^{-1})	v_{mRNA} (mmol·gDW^{-1}·h^{-1})	$cost_{AA}$ ($0.27/v_{AA}$)	$cost_{nucl\text{-}sRNA}$ ($0.27/v_{sRNA}$)	$cost_{nucl\text{-}mRNA}$ ($0.27/v_{mRNA}$)
42°C glucose	0.66	4.536	2.678	2.211	0.060	0.101	0.122
Acetate	0.30	3.091	1.509	1.199	0.087	0.179	0.225
Chemostat μ = 0.12	0.12	4.536	2.678	1.256	0.060	0.101	0.215
Chemostat μ = 0.2	0.20	4.536	2.678	1.592	0.060	0.101	0.170
Chemostat μ = 0.35	0.35	4.536	2.678	1.923	0.060	0.101	0.140
Chemostat μ = 0.5	0.50	4.536	2.678	2.098	0.060	0.101	0.129
Fructose	0.65	4.343	2.535	2.106	0.062	0.106	0.128
Fumarate	0.42	4.229	2.360	1.827	0.064	0.114	0.148
Galactose	0.26	4.480	2.629	1.734	0.060	0.103	0.156
Glucosamine	0.46	4.344	2.537	1.974	0.062	0.106	0.137
Glucose	0.58	4.536	2.678	2.161	0.060	0.101	0.125
Glycerol	0.47	4.651	2.796	2.140	0.058	0.097	0.126
Glycerol + AA	1.27	7.033	7.965	5.941	0.038	0.034	0.045
LB	1.90	7.048	34.355	17.428	0.038	0.008	0.015
Mannose	0.47	4.330	2.525	1.976	0.062	0.107	0.137
Osmotic-stress glucose	0.55	4.536	2.678	2.139	0.060	0.101	0.126
pH6 glucose	0.63	4.536	2.678	2.194	0.060	0.101	0.123
Pyruvate	0.40	3.605	1.930	1.542	0.075	0.140	0.175
Succinate	0.44	4.282	2.456	1.905	0.063	0.110	0.142
Xylose	0.55	4.386	2.568	2.069	0.062	0.105	0.131

Supplementary Table 5. Class 1 and Class 2 codons.

Codon class	Codons
Class 1	GCA, GCG, GCU, GCC, CGC, CGU, CGA, CGG, AGA, AGG, AAC, AAU, GAC, GAU, UGC, UGU, CAA, CAG, GAG, GAA, GGA, GGU, GGC, CAC, CAU, AUC, AUU, AUA, CUU, CUC, CUA, UUA, AAG, AAA, AUG, UUU, UUC, CCC, CCA, UCA, AGU, AGC, UCC, ACC, ACA, UGG, UAU, UAC, GUG, GUA, GUC
Class 2	GGG, CUG, UUG, CCG, CCU, UCG, UCU, ACU, ACG, GUU

Supplementary References

1. Scott, M., Klumpp, S., Mateescu, E. M. & Hwa, T. Emergence of robust growth laws from optimal regulation of ribosome synthesis. *Mol. Syst. Biol.* **10**, 747 (2014).
2. Scott, M., Gunderson, C. W., Mateescu, E. M., Zhang, Z. & Hwa, T. Interdependence of cell growth and gene expression: origins and consequences. *Science* **330**, 1099–102 (2010).
3. Klumpp, S., Scott, M., Pedersen, S. & Hwa, T. Molecular crowding limits translation and cell growth. *Proc. Natl. Acad. Sci. U. S. A.* **110**, 16754–9 (2013).
4. Schmidt, A. *et al.* The quantitative and condition-dependent Escherichia coli proteome. *Nat. Biotechnol.* **34**, 104–110 (2016).
5. Ehrenberg, M. & Kurland, C. G. Costs of Accuracy Determined by a Maximal Growth Rate Constraint. *Q. Rev. Biophys.* **17**, 45–82 (1984).
6. Dai, X. *et al.* Reduction of translating ribosomes enables Escherichia coli to maintain elongation rates during slow growth. *Nat. Microbiol.* **2**, 16231 (2016).
7. Mori, M., Schink, S., Erickson, D. W., Gerland, U. & Hwa, T. Quantifying the benefit of a proteome reserve in fluctuating environments. *Nat. Commun.* **8**, 1–8 (2017).
8. Feist, A. M. *et al.* A genome-scale metabolic reconstruction for Escherichia coli K-12 MG1655 that accounts for 1260 ORFs and thermodynamic information. *Mol. Syst. Biol.* **3**, 121 (2007).
9. Gromadski, K. B., Wieden, H.-J. & Rodnina, M. V. Kinetic mechanism of elongation factor Ts-catalyzed nucleotide exchange in elongation factor Tu. *Biochemistry* **41**, 162–9 (2002).
10. Bennett, B. D. *et al.* Absolute metabolite concentrations and implied enzyme active site occupancy in Escherichia coli. *Nat. Chem. Biol.* **5**, 593–9 (2009).
11. Valgepea, K., Adamberg, K., Seiman, A. & Vilu, R. Escherichia coli achieves faster growth by increasing catalytic and translation rates of proteins. *Mol. Biosyst.* **9**, 2344 (2013).
12. Dong, H., Nilsson, L. & Kurland, C. G. Co-variation of tRNA Abundance and Codon Usage inEscherichia coliat Different Growth Rates. *J. Mol. Biol.* **260**, 649–663 (1996).
13. Forchhammer, J. & Lindahl, L. Growth rate of polypeptide chains as a function of the cell growth rate in a mutant of Escherichia coli 15. *J. Mol. Biol.* **55**, 563–8 (1971).
14. Skjold, A. C., Juarez, H. & Hedgcoth, C. Relationships among deoxyribonucleic acid, ribonucleic acid, and specific transfer ribonucleic acids in Escherichia coli 15T- at various growth rates. *J. Bacteriol.* **115**, 177–187 (1973).
15. Hui, S. *et al.* Quantitative proteomic analysis reveals a simple strategy of global resource allocation in bacteria. *Mol. Syst. Biol.* **11**, 784 (2015).
