# Supplementary material for: The protein translation machinery is expressed for maximal efficiency in Escherichia coli
Source: Nat Commun. 2020 Oct 16;11:5260. doi: 10.1038/s41467-020-18948-x (PMC7568582; doi:10.1038/s41467-020-18948-x)
Supplement: Supplementary file 3 — Description of Additional Supplementary Files [file 41467_2020_18948_MOESM3_ESM.pdf]

Description of Additional Supplementary Files

File Name: Supplementary Data 1
Description: All reactions and parameters included in the model (see also Supplementary Data 4 and 5).

File Name: Supplementary Data 2
Description: Molecular weights and alternative cost measures for all model components.

File Name: Supplementary Data 3
Description: SBML Model description (SBML Level 3 version 2). To help the reader understand and explore the mechanics of the model described in the main article, we provide a comprehensive kinetic model, fully annotated and compliant with SBML Level 3 version 2. This model includes all species, reactions, parameters and initial values to perform steady-state and time-course analysis in a glucose minimal medium at a specific growth rate (0.58 h^{-1}).

File Name: Supplementary Data 4
Description: GAMS input file for the optimization problem, including the model and its parameterization; protein production requirements are set to those for growth on a minimal glucose medium.

File Name: Supplementary Data 5
Description: Source data for Figures 2-5.
